# Structure-based discovery of two antiviral inhibitors targeting the NS3 helicase of Japanese encephalitis virus

**DOI:** 10.1038/srep34550

**Published:** 2016-09-29

**Authors:** Jin’e Fang, Huan Li, Dexin Kong, Shengbo Cao, Guiqing Peng, Rui Zhou, Huanchun Chen, Yunfeng Song

**Affiliations:** 1 State Key Laboratory of Agricultural Microbiology, Huazhong Agricultural University, Wuhan, China; 2 Key Laboratory of Veterinary Diagnostic Products, College of Veterinary Medicine, Huazhong Agricultural University, Wuhan, China; 3 College of Informatics, Huazhong Agricultural University, Wuhan, China

## Abstract

Japanese encephalitis virus (JEV) is a flavivirus that threatens more than half of the world’s population. Vaccination can prevent the disease, but no specific antiviral drug is yet available for clinical therapy, and the death rate caused by JEV can reach as high as 60%. The C-terminus of non-structural protein 3 (NS3) of flavivirus encodes helicase and has been identified as a potential drug target. In this study, high throughput molecular docking was employed to identify candidate JEV NS3 helicase inhibitors in a commercial library containing 250,000 compounds. Forty-one compounds were then tested for their ability to inhibit NS3 activity. Two compounds inhibited unwinding activity strongly but had no effect on the ATPase activity of the protein. Western blots, IFA, and plaque reduction assays demonstrated that both compounds inhibited the virus in cell culture. The EC50s of the two compounds were 25.67 and 23.50 μM, respectively. Using simulated docking, the two compounds were shown to bind and block the NS3 RNA unwinding channel, consistent with the results of the enzyme inhibition tests. The atoms participating in intramolecular interaction were identified to facilitate future compound optimization.

Japanese encephalitis virus (JEV), a member of the genus *Flavivirus* in the family *Flaviviridae*, was first reported in Japan in the 1870s. JEV has spread to most countries in south, east and south-east Asia^1^, and since the 1990s, it has spread to Australia^2^, Pakistan^3^, and Saipan^4^ and now threatens more than three billion people^5^. JEV can cause acute viral-encephalitis in 0.1–2% of people infected, with a fatality rate of 10–50%, and of those that survive, half have severe neurologic sequelae including persistent motor defects and severe cognitive and language impairments^6^. Although vaccines for prevention of JEV have been available for many years^7^,^8^, 50,000 cases and 10,000 deaths still occur annually. Specific anti-JEV agents that can reduce the death toll and neurological sequelae are urgently needed^9^. Recently, several flavivirus non-structural (NS) proteins, such as NS2B-NS3, NS3 helicase, NS4B, NS5 methyltransferase, and NS5 RNA-dependent RNA polymerase (RdRp), have been identified as potential anti-viral drug targets^10^,^11^,^12^. Many compounds have an inhibitory effect on the virus, raising hopes that new treatments are on the horizon for prevention and treatment of flavivirus-associated diseases^13^.

NS3 of JEV is a bifunctional protein possessing both protease and RNA helicase activities. The NS3 N-terminal region, spanning 179 amino acids, interacts with NS2B to cleave the polyprotein precursor into individual proteins^10^. The C-terminal fragment of NS3 behaves as an ATP-dependent RNA helicase and unwinds the double-stranded RNA genome during virus replication^14^. Because the NS3 helicase of flavivirus plays an essential role during the viral life circle, it has attracted attention as a potential drug target^15^. Although the flavivirus NS3 helicase and human DDX3 proteins share some identical residues^16^, a markedly divergent sequence is found outside of the conserved regions, making it possible to design virus-specific inhibitors. Several antiviral inhibitors against dengue virus helicase have been identified, such as Suramin^17^, ST-610^18^, and ivermectin^19^. In contrast, efforts to identify agents effective against Japanese encephalitis virus and West Nile virus have been very limited.

## Results

### Virtual screening of NS3 helicase inhibitors

Virtual screening was conducted using GOLD Suite 5.1 (Genetic Optimization for Ligand Docking). Compounds from the library (Specs) were docked to the full structure of NS3 to predict binding ability. Interaction analyses were performed using Discovery Studio Visualizer 3.1 (Accelrys, San Diego, CA, USA). Forty-one compounds from the top-scoring molecules were selected for further evaluation. Compound profiles are provided in Supplementary Table S1.

### Inhibition of NS3 enzyme activities

**Figure 1:**
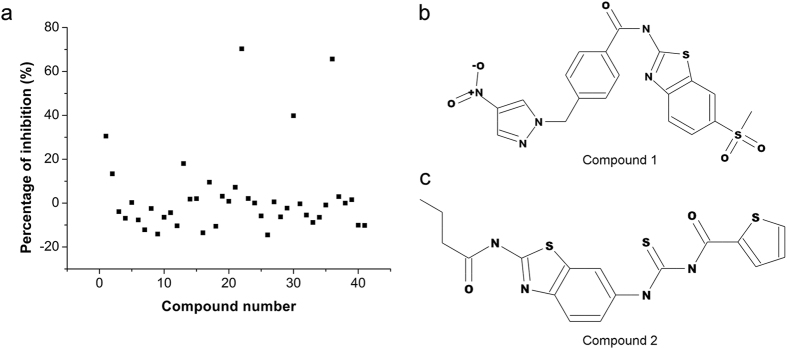
Helicase inhibitor screening for selected compounds. Panel a shows the inhibitory effect of each compound on NS3 unwinding activity. Each dot represents one compound. Two compounds exhibit inhibition levels over 60% and were designated compound 1 and compound 2. Panel b shows the two-dimensional structure of compound 1, and panel c compound 2.

Screening for helicase inhibitors. The selected compounds were tested for an inhibitory effect on NS3 unwinding activity using a fluorescence resonance energy transfer (FRET) assay. Four compounds inhibited unwinding activity more than 30%, and two of these inhibited by more than 60% (Fig. 1a). These two compounds, designated compound 1 and 2, were then selected for subsequent experiments. These compounds are classified into heterocyclic organic compound. Their structures are shown in Fig. 1b,c. The Specs ID number for these two compounds were AK-968/40733793 and AP-970/43253453, respectively.

### Quantification of inhibitory effects on NS3 helicashe unwinding activity

**Figure 2:**
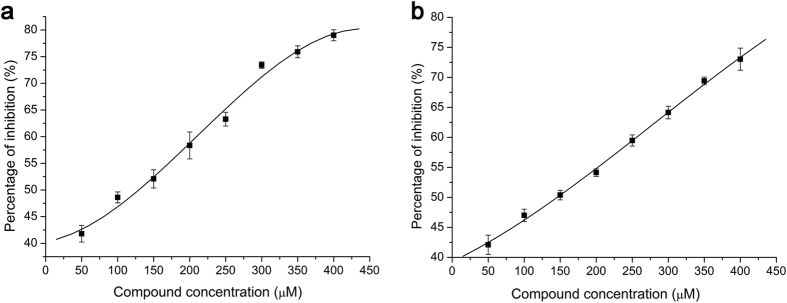
Quantification of inhibitory effects on unwinding activity. Compounds 1 (a) and 2 (b) in different concentrations were added to the unwinding reaction. Percentage of inhibition was calculated for every concentration and trend lines were fitted by non-linear regression.

To evaluate the inhibitory effect on NS3 helicase unwinding activity provided by the two hit candidates, different concentrations of each compound were added to the unwinding reaction mixture, and the percentage of inhibition was calculated based on observed fluorescence. Both compounds showed concentration-dependent inhibition, with inhibitory levels reaching as high as 79% for compound 1 and 73% for compound 2 (Fig. 2a,b). The median inhibitory concentrations (IC50) were 148 μM (compound 1) and 150 μM (compound 2), estimated by non-linear regression.

### Effect on the ATPase activity of NS3 helicase

**Figure 3:**
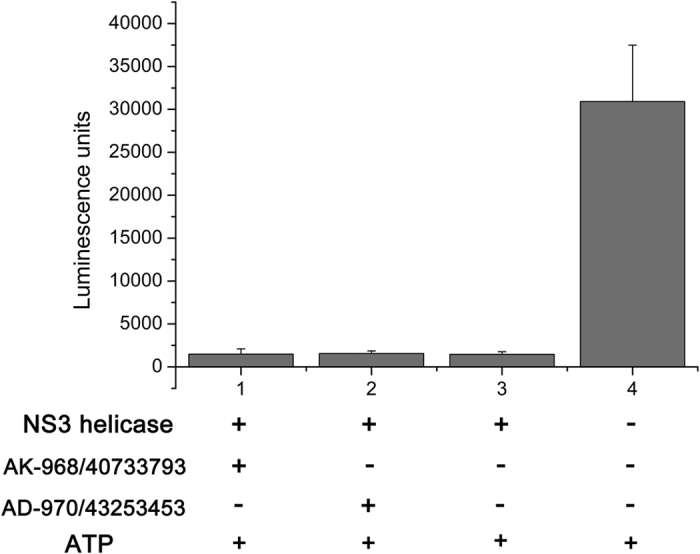
Tests for inhibition of ATPase activity. Compounds 1 (column 1) and 2 (column 2) were added to the ATPase reaction mixture containing recombinant NS3 helicase protein. After incubation, luminescence was measured to determine levels of hydrolyzed ATP. Samples without compound (column 3) and without compound and NS3 helicase (column 4) were used as controls. Bars represent the standard deviation from three replicates.

Compounds 1 and 2 were added to an ATPase reaction mixture to a final concentration of 200 μM. After 30 min, residual ATP was detected using a luminescence reagent (Kinase-Glo Plus reagent, Promega). As expected, the reaction without NS3 protein exhibited high luminescence (Fig. 3 column 4). The reaction with NS3 and ATP as an ATPase positive control, exhibited very low luminescence (Fig. 3 column 3). ATP levels in reactions that included either compound were statistically indistinguishable from the ATPase positive control (P > 0.05) (Fig. 3 columns 1 and 2), indicating that these compounds at 200 μM do not inhibit the ATPase activity of NS3 helicase.

### Evaluation of antiviral activity

**Figure 4:**
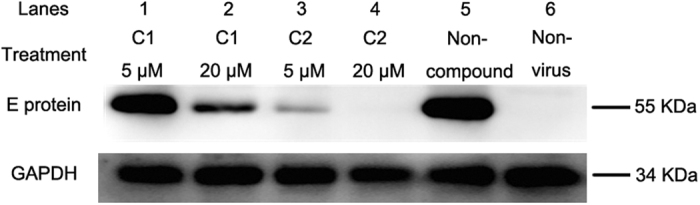
Identification of antiviral effects by Western blot. JEV was propagated in the presence of each of the compounds at 5 μM (lanes 1 and 2) and 20 μM (lanes 3 and 4). JEV infected cells without compound (lane 5) and mock-infected cells (lane 6) were used as controls. Envelope protein expression was evaluated by Western blot using anti-envelope monoclonal antibody. GAPDH served as a control for the amount of protein in each lane. Compound 1 inhibited envelope protein expression at 20 μM (lane 3) compared to the JEV-infected control (lane 5). Compound 2 significantly inhibited expression at 5 μM (lane 2). Envelope protein expression was completely inhibited by 20 μM of compound 2 (lane 4). C1 and C2 in the figure indicate compound 1 and 2, respectively.

Identification of antiviral effects by Western blot. JEV was propagated in BHK-21 cells for 48 h in the presence of the compounds at concentrations of 5 and 20 μM. E protein expression was detected by Western blot, with GAPDH serving as a loading control. The protein expression level was estimated by measuring band intensity with the image analysis application ImageJ. The expression of E protein was normalized by the expression of GAPDH and the changes of E expression were calculated relative to the non-compound treated group. At 20 μM, compound 1 (Fig. 4, lane 2) and compound 2 (Fig. 4, lane 4) reduced the expression of E protein by 70.1% and 94.8%, respectively. Additionally, compound 2 inhibited 86.3% of E protein expression at 5 μM (Fig. 4, lane 3). GAPDH expression was not inhibited by the compounds, demonstrating that they had no effect on cell growth. E protein expression was decreased by treatment with either compound at concentrations of 5 or 20 μM, indicating that they both inhibited JEV propagation.

### Identification of antiviral effects by indirect immunofluorescence assay (IFA)

**Figure 5:**
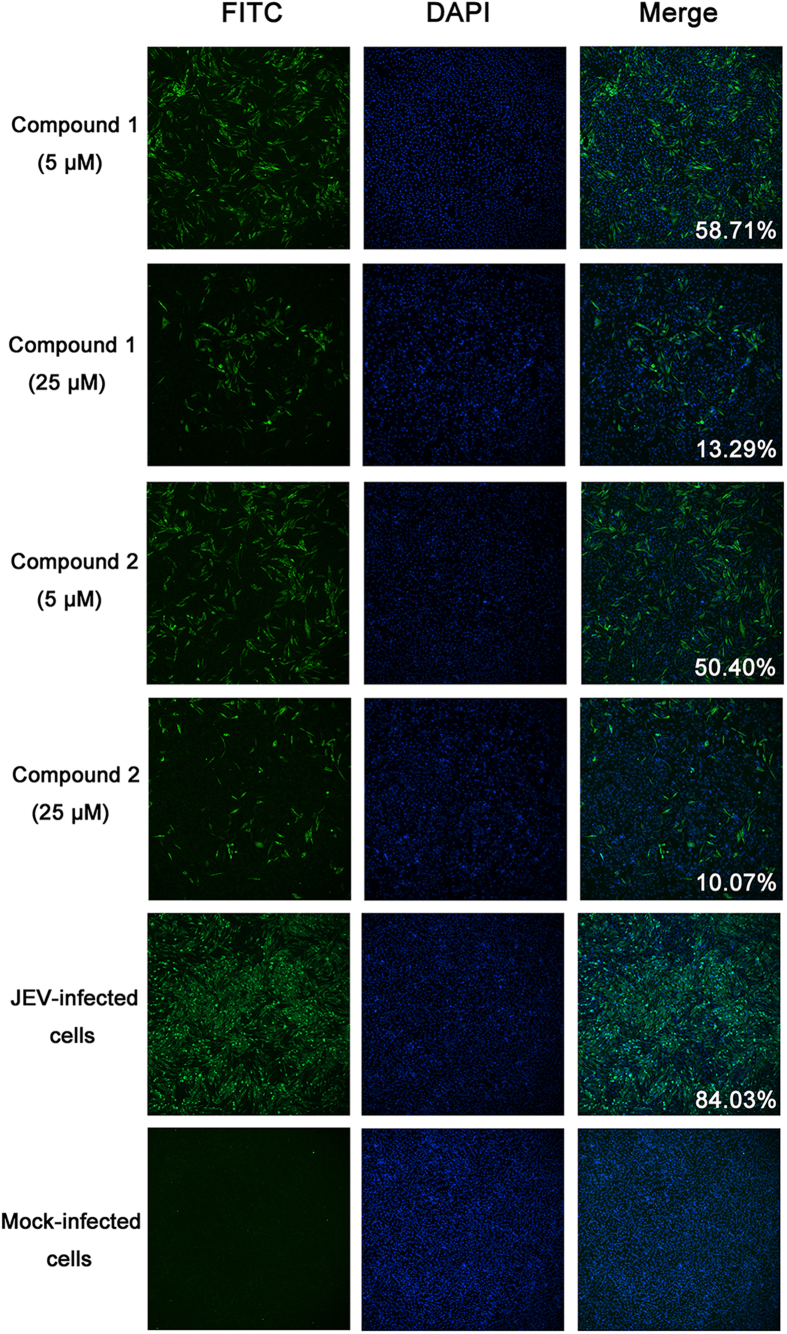
Evaluation of antiviral effect by indirect immunofluorescence assay (IFA). JEV was cultured in BHK-21 cells with the compounds of interest at 5 or 25 μM. Mock-infected cells and JEV-infected cells without compound were used as controls. The expression of JEV NS5 was detected by IFA. The percentage of positive cells is shown in the merged panels.

JEV was propagated in BHK-21 cells for 48 h with compound at 5 and 25 μM. Levels of intracellular virus were measured by IFA with a NS5-specific monoclonal antibody. As expected, mock-infected cells had no NS5 expression, while 84.03% of JEV-infected cells were positive for NS5 expression. Both compounds inhibited JEV replication at each concentration; the levels of JEV-positive cells were 58.71% and 50.40% in the presence of 5 μM compounds 1 and 2 respectively, and 13.29% and 10.07% in the presence of 25 μM compounds 1 and 2, respectively (Fig. 5).

### Identification of antiviral effect by plaque reduction assay

**Figure 6:**
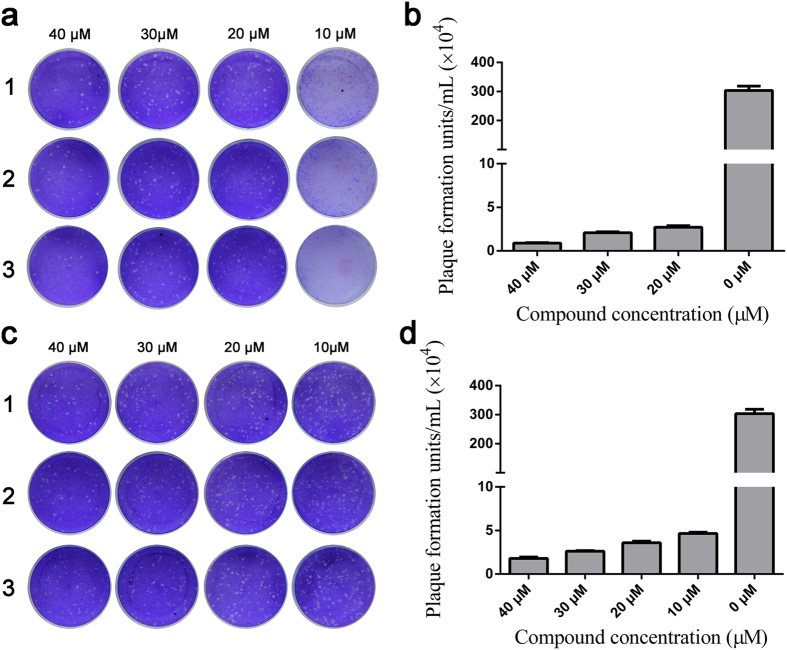
Plaque reduction assay. JEV was cultured in BHK-21 cells in presence of different compound concentrations. Panel a is the plaque reduction assay for compound 1 and panel c for compound 2. The corresponding plaque formation units are shown in panels b and d. The virus titer in the untreated control (no compound) is shown as 0 μM. Bars represent the standard deviation of mean.

JEV was propagated in BHK-21 cells in the presence of compounds 1 or 2 at concentrations of 10, 20, 30, and 40 μM. After 48 h incubation, virus titers were determined by plaque assay. The titer in the untreated (control) group was 3.03 × 10^6^ plaque formation units (PFU)/mL. In compound-treated cells, plaque numbers were all significantly reduced (P < 0.001) compared to the untreated control group. Average virus titers in the 20, 30, and 40 μM compound 1 treated samples were 2.7 × 10^4^, 2.1 × 10^4^, and 8.9 × 10^3^ PFU/mL, respectively (Fig. 6b). Average virus titers in the 10, 20, 30, and 40 μM compound 2 treated samples were 4.65 × 10^4^, 3.6 × 10^4^, 2.6 × 10^4^, and 1.8 × 10^4^ PFU/mL, respectively (Fig. 6c).

### Determination of 50% effective concentration (EC50) and 50% cytotoxicity concentration (CC50)

**Figure 7:**
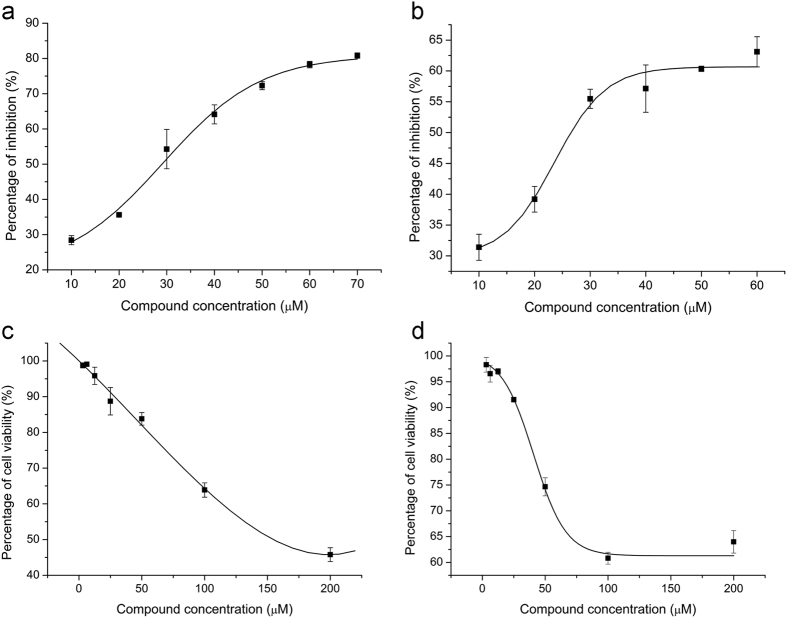
50% effective concentration (EC50) and 50% cytotoxicity concentration (CC50) for each compound. The percentage of virus inhibition was determined by comparing the cell viability in JEV-infected and compound-treated samples with mock-infected controls. Panel a shows the inhibition curve for compound 1 and b for compound 2. The percentage of cell viability was determined in BHK-21 cells incubated with different compound concentrations. Panel c shows the cytotoxicity curve for compound 1 and panel d for compound 2. All fitted curves were generated by non-linear regression. Bars represent the standard deviation of mean.

To determine the EC50, each compound was added to JEV-infected cells over a range of concentrations. Cell viability was measured 120 h post infection and percent inhibition was calculated. Both compounds exhibited dose-dependent inhibition of JEV (Fig. 7a,b). The EC50s of compounds 1 and 2 were estimated at 25.67 and 23.50 μM, respectively. To determine the CC50, each compound was added to BHK-21 cells at different concentrations and the cell viability tested after 120 h. Both compounds showed low cytotoxicity. The CC50 of compound 1 was estimated at 168.07 μM (Fig. 7c), while cell viability was more than 60% in the presence of 200 μM compound 2 (Fig. 7d).

### Potential binding sites

**Figure 8:**
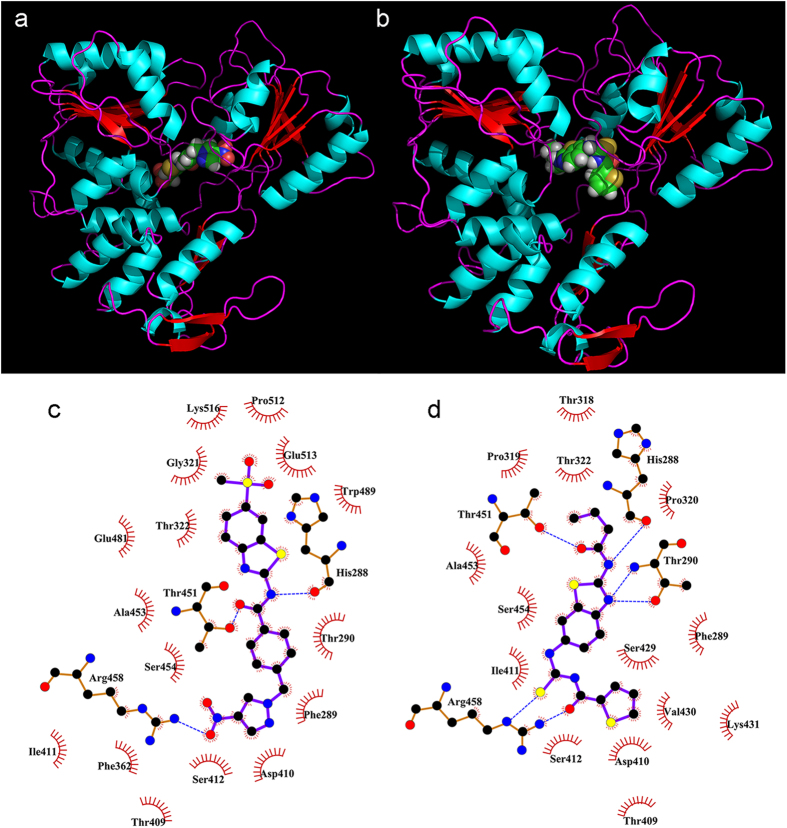
Simulated binding to NS3 helicase. Both compounds exhibit high binding scores in the RNA unwinding channel contacting domains I and II. Panel a shows the predicted binding mode for compound 1 and panel b for compound 2. The proteins are represented using ribbon diagrams and the compounds are drawn using space-filling models. Panels c (compound 1) and d (compound 2) show the intermolecular forces between compounds and protein. The amino acids involved in intermolecular H-bonds (blue dashes) are represented using ball-and-stick models. Non-ligand residues involved in hydrophobic contacts are labeled with name and amino acid residue number (semi-circular glyphs).

Potential binding sites were examined in detail using molecular simulation to dock each compound to the NS3 helicase protein. Both compounds exhibited high binding scores in the unwinding channel contacting domains I and II (Fig. 8a,b). Compound 1 interacted with NS3 via three hydrogen bonds and hydrophobic forces, while compound 2 interacted with the binding site via six hydrogen bonds and hydrophobic forces (Fig. 8c,d). The amino acid residues His288, Thr451, and Arg458 in NS3 were shown as common hydrogen bond donors in both of the compounds binding.

## Discussion

In recent years, antiviral drugs have become an important strategy for preventing and treating viral diseases. Several diseases caused by flaviruses, such as West Nile virus (WNV), Dengue virus (DENV) and JEV, threaten public health but still lack specific antiviral therapies. Some compounds have been found to have antiflavivirus effects, targeting the viral protease, helicase, or RdRp. A tripeptide (phenacetyl-KKR-Aldehyde) can inhibit the activity of WNV NS2B-NS3 protease (Ki 9 nM) and thus has a highly inhibitory effect on the virus in cells (EC50 1.6 μM)^20^. Ivermectin, a widely used antihelminthic drug, was proven to have high inhibitory effects on yellow fever virus (YFV), DENV, JEV, and tick-borne encephalitis viruses (TBEV) by blocking the viral helicase^19^. A small-molecule compound, ST-610, was shown to inhibit the unwinding activity of DENV helicase but not the ATPase activity. The compound can inhibit all four types of DENV at high efficiency^18^. Additional anti-flavivirus inhibitor screening is required to provide new candidates for drug development.

The NS3 helicase of flavivirus is classified as a superfamily 2 helicase, also termed DExD(/H)-box helicase^21^. Helicases in this family consist of three domains that are responsible for the NTPase and helicase activities^22^. Domains I and II form a RecA-like domain containing the motifs essential for RNA-binding and ATP hydrolysis. The domain 3 with domains 1 and 2 form a channel for single-stranded RNA binding and double-stranded RNA unwinding^23^. For the purposes of drug discovery, several general conceptual strategies have been proposed to inhibit NS3 helicase, including inhibiting NTP-binding, NTP-hydrolysis, RNA-binding, RNA-unwinding, and the coupling of hydrolysis to unwinding^16^. In this study, both compounds of interest inhibit unwinding activity in a dose-dependent manner (Fig. 2), but fail to inhibit the ATPase activity of NS3 helicase (Fig. 3). These results suggest that the compounds bind to the unwinding channel but not the ATP hydrolysis cleft, and are consistent with binding simulated by molecular docking (Fig. 8a,b). By focusing on the atoms involved in the protein-ligand interactions (Fig. 8c,d), it may be possible to modify compound to optimize binding ability.

Lead compound discovery in drug development is both time consuming and costly. The typical rate at which interesting candidates are “hit” in high throughput screening is around 0.01–0.1%. As one method of rational drug design, high throughput docking is gaining increasing usage in drug discovery, coupled with rapid developments in structural biology^24^. By testing high-scoring molecules in virtual screening, it is possible to increase “hit” rates to high levels in some cases^25^. Before commencing our study, we verified the accuracy of different docking software by docking adenosine-5′-diphosphate (ADP) to dengue virus NS3 helicase, and then comparing the predicted conformations with crystal structure data (PBD code 2JLZ). Only the prediction generated by the GOLD package was similar, and we therefore selected GOLD as the scoring method in this study. After the initial screen by high throughput docking, we tested the 41 high-scoring compounds and identified two with antiviral effects, yielding a hit rate of 4.9%.

In tests to quantify inhibitory effects, compounds 1 and 2 had similar EC50s (25.67 μM and 23.50 μM, respectively). But both compounds exhibited viral inhibition of over 50% at concentrations of 20 μM as determined by Western blot and plaque reduction. At this concentration, very few or no viral proteins could be detected by Western blot (Fig. 4). In plaque reduction assays, virus titers after treatment with 20 μM compounds were 2.7 × 10^4^ PFU/mL (compound 1) and 3.6 × 10^4^ PFU/mL (compound 2) (Fig. 6). In contrast, virus titers in the untreated control reached 3.03 × 10^6^ PFU/mL, more than 80 times higher. Thus, the actual EC50 of the compounds should be less than 20 μM.

In summary, we found two compounds that target the JEV helicase and inhibit the virus at micromolar levels. These two compounds were predicted to bind in the RNA channel of NS3 helicase. Because the RNA binding cleft is a large cavity, the opportunity exists to optimize the compounds by a structural-based drug design.

## Methods

### Plasmid, cells, virus strain, and compounds

The NS3 helicase gene (amino acid residues 180-619 of NS3) was cloned from the JEV P3 strain (GenBank accession number U47032.1) and then subcloned into prokaryotic expression vector pET-30a (Merck, Darmstadt, Germany). The expression plasmid was transformed into *E.coli* BL21 (DE3) pLysS competent cells (Promega, Madison, WI, USA) for protein expression. Baby hamster kidney 21 (BHK-21) cells were cultured in growth medium at 37 °C with 5% CO2 and 80–95% humidity. The growth medium was Dulbecco’s modified Eagle’s medium (DMEM) (Sigma-Aldrich, St. Louis, MO, USA) supplemented with 10% fetal calf serum (FCS) (Invitrogen, Grand Island, NY, USA), 100 U/mL of penicillin, and 100 μg/mL of streptomycin (Sigma-Aldrich). The JEV P3 strain was propagated in BHK-21 cells with maintenance medium containing 1% FCS, 100 U/mL of penicillin, and 100 μg/mL of streptomycin. The compounds were purchased from Specs (Delft, Netherlands) and dissolved in dimethyl sulphoxide (DMSO) (Applichem, Darmstadt, Germany) to 10 mM.

### High throughput virtual screening of NS3 helicase inhibitors

The crystal structure of NS3 helicase (PDB code 2Z83) and a database containing about 250,000 compounds (Specs, Delft, Netherlands) were used to conduct high throughput virtual screening. The amino acid residues spanning positions 245-252 and 413-415 were not visible in the NS3 helicase structure because they were disordered in the protein. These two regions were reconstructed by homology modeling based on the corresponding structures in dengue virus NS3 helicase (PDB code 2JLS). The 3-dimensional conformations of the compounds were generated by OMEGA (Ver. 2.4.3) (OpenEye Scientific Software, Santa Fe, NM, USA). The compounds were docked freely to the whole structure of NS3 helicase using GOLD suite 5.1 (CCDC, Cambridge, UK). No water was present in the binding sites. The genetic algorithm (GA) parameter was set to 100% search efficiency and the best pose was saved. The fitness of compounds to protein was scored by GoldScore. Two hundred of the top scoring compounds were selected for interaction analysis. The best candidates were selected using a combination of scoring, fitness, and compound similarity.

### Expression, purification, and identification of NS3 helicase protein

The expression, purification, and identification of NS3 helicase were performed as previously described^26^. Briefly, the pET-30a construct containing the NS3 helicase gene fragment was transformed into *E.coli* BL21 (DE3) pLysS competent cells, and expression was induced by 0.5 mM Isopropyl β-D-1-thiogalactopyranoside (IPTG) (Sigma-Aldrich). Recombinant NS3 helicase was purified by Ni^2+^ affinity column chromatography, followed by gel filtration chromatography. The expressed protein was identified by Western blot with His-tag Mab (Proteintech, Chicago, IL, USA) and NS3-specific Mab (prepared in our laboratory) as previously described^26^.

### NS3 helicase inhibition assay

The ability of each compound to inhibit NS3 helicase was evaluated by a fluorescence resonance energy transfer (FRTE) assay as previously described^26^. The reaction mixture, containing 3 μM NS3 helicase, various concentrations of compound, 200 nM of substrate DNA, and 1.2 μM of capture strand DNA, was aliquoted into a 96-well black plate (Jet Bio-Filtration, Guangzhou, China). Three independent reactions were conducted for each test. The reaction was initiated by adding 2.5 mM ATP per well. After incubation at 37 °C for 60 min, the fluorescence value (Ex 635 nm and Em 670 nm) of each well was measured using an Infinite 200 PRO multimode reader (Tecan, Männedorf, Switzerland). The percentage of inhibition was calculated as: 1 − ((Fx − Fn)/(Fp − Fn)), where Fx is the fluorescence value of an experimental sample, Fn is the fluorescence value of a control sample without nucleic acid substrate, and Fp is the fluorescence value of a control sample without compound.

### Effect on the ATPase activity of NS3 helicase

The effect of each compound on ATPase activity was tested using a nonradioactive method as previously described^26^. The compounds (final concentration 200 μM) were added to the reaction mixture that had been aliquoted previously into a 96-well black plate (Jet Bio-Filtration). The reaction mixture comprised 3 μM of recombinant NS3 helicase and 100 μM ATP in ATPase reaction buffer (20 mM Tris-HCl pH 8.0, 50 mM NaCl, 10 mM MgCl_2_). Three reactions were conducted for each test. After incubation at 37 °C for 30 min, an equal volume of Kinase-Glo Plus reagent (Promega) was added to each well to quantitate the remaining ATP. The luminescence value of each well was tested by an Infinite 200 PRO multimode reader.

### Identification of antiviral effects by Western blot

After BHK-21 cells were infected with JEV (MOI 0.01), compounds were added to culture medium at final concentrations of 5 and 20 μM, and cells were incubated for 48 h. Expression of JEV envelope (E) protein was detected by Western blot. Total cellular protein was mixed with 5 × SDS-loading buffer and boiled for 10 min. The protein was electrophoresed on 12% SDS-PAGE and transferred to a nitrocellulose filter membrane (Millipore, Darmstadt, Germany). The membrane was washed with TBST and blocked for 2 h with blocking buffer (TBS containing 1.5% BSA and 1.5% powdered skim milk) at 37 °C. The protein was reacted with JEV envelope protein-specific monoclonal antibody (1:500) or GAPDH-specific monoclonal antibody (1:1000; Proteintech) at 37 °C for 2 h. After three washes with TBST, the membrane was stained with horseradish-peroxidase-conjugated goat anti-mouse IgG (Boster, Wuhan, China) at 37 °C for 1 h. Protein blots were developed using SuperSignal West Pico Chemiluminescent Substrate (ThermoFisher Scientific, Rockford, IL, USA) following the manufacturer’s instructions.

### Identification of the antiviral effect by indirect immunofluorescence assay (IFA)

Compounds were added to JEV-infected cells at concentrations of 5 or 25 μM. After incubation for 48 h, virus replication was evaluated by IFA. Cells were fixed with formaldehyde for 5 minutes, then anti-NS5 Mab (1:500)^27^ and FITC-conjugated goat anti-mouse antibody (Boster) were added to cells as primary and secondary antibodies. Finally, the cells were stained with DAPI (Sigma-Aldrich) and imaged by a fluorescence microscope (IX83; Olympus, Tokyo, Japan). The number of JEV-positive or negative cells in each visual field was counted using cellSens Dimension (Ver. 1.7.1) (Olympus).

### Identification of antiviral effects by plaque reduction assay

BHK-21 cells in 96-well were infected with JEV at MOI 0.01 in the presence of different concentrations of compound (10, 20, 30, and 40 μM). Each compound was represented in three wells at each concentration. Forty-eight hours post-infection, viruses in each triplicate group were harvested by three cycles of freezing/thawing, and then combined in one tube. JEV titers were measured using a plaque assay as previously described^28^. Briefly, BHK-21 cells in 12-well plates were inoculated with 50 μL of diluted virus (20×) in three technical replicates. For the non-compound control, 10-fold serially diluted virus was added to the BHK-21 cells. After adsorption at 37 °C for 60 min, cells were washed with phosphate buffered saline (PBS) three times and overlaid with DMEM containing 2% FCS and 1% carboxymethylcellulose (Sigma-Aldrich). After 5 days incubation, the cells were fixed with 10% formalin and stained with 0.5% crystal violet. The average plaque number was calculated ± standard deviation (SD).

### Determination of EC50 (50% of effective concentration)

BHK-21 cells were seeded into 96-well white plates (Corning, Tewksbury, MA, USA) at 10,000 cells per well. Twelve hours later, the growth medium was replaced with maintenance medium containing 0.01 MOI JEV and various concentrations of compounds. Mock-infected cells and JEV-infected cells without compound treatment were used as controls. The cells were incubated for 120 h, then cell viability was measured by a cell viability test reagent (Celltiter-Glo, Promega) as described previously^29^. Three repetitions were conducted for each test and the average luminescence value was calculated. The percentage of CPE inhibition was calculated as 1 − (Lx − Lv)/(Lc − Lv), where Lx is the luminescence value of an experimental sample, Lv is the luminescence value of a virus-infected control, without compound, and Lc is the luminescence value of a mock-infected sample. The EC50 values were estimated using nonlinear regression analysis.

### Compound cytotoxicity

BHK-21 cells were seeded into 96-well white plates at 10,000 cells per well. After incubation for 12 h to allow cell attachment, different concentrations of compounds were added to the cells, with three identical reactions at each concentration. Cell viability was measured after 120 h incubation. The percentage of cell viability was calculated as Lx/Lc, where Lx is the luminescence value of an experimental sample, and Lc is the luminescence value of a compound-free control. The 50% cytotoxicity concentration (CC50) was estimated by nonlinear regression analysis.

### Binding site prediction

The binding of the two compounds of interest to NS3 helicase was simulated using GOLD Suite 5.1. The 3-dimensional conformations of the compounds were generated by OMEGA (Ver. 2.4.3). The compounds were docked to the whole protein and the most probable binding pattern was recorded using the PyMol molecular visualization system. The interaction analyses were performed by Ligplot+^30^.

### Statistical analysis

In the ATPase inhibition, unwinding inhibition, plaque reduction, EC50 and CC50 assay, each test was conducted in triplicate, and the data are expressed as mean ± standard deviation (SD). The differences between groups were evaluated using Student’s t-test. P < 0.05 was considered as statistically significant, and P < 0.001 was extremely significant.

## Additional Information

**How to cite this article**: Fang, J. *et al.* Structure-based discovery of two antiviral inhibitors targeting the NS3 helicase of Japanese encephalitis virus. *Sci. Rep.* **6**, 34550; doi: 10.1038/srep34550 (2016).

## Supplementary Material

Supplementary Information
