# Supplementary material for: Structure-based discovery of two antiviral inhibitors targeting the NS3 helicase of Japanese encephalitis virus
Source: Sci Rep. 2016 Sep 29;6:34550. doi: 10.1038/srep34550 (PMC5041104; doi:10.1038/srep34550)
Supplement: Supplementary Information [file srep34550-s1.pdf]

Structure-based discovery of two antiviral inhibitors targeting the NS3 helicase of Japanese encephalitis virus

Jin'e Fang[1,2], Huan Li[2], Dexin Kong[1,3], Shengbo Cao[1,2], Guiqing Peng[1,2], Rui Zhou[1,2], Huanchun Chen[1,2], Yunfeng Song[1,2*]

1. State Key Laboratory of Agricultural Microbiology, Huazhong Agricultural University, Wuhan, China

2. Key Laboratory of Veterinary Diagnostic Products, College of Veterinary Medicine, Huazhong Agricultural University, Wuhan, China

3. College of Informatics, Huazhong Agricultural University, Wuhan, China

Supplementary table S1

IDNUMBER	WEIGHT	FORMULA	SMILES
AQ-364/41885139	490.59	C30H34O6	C=CCOC4C(O)C(O)C(OCc1ccccc1)C(OCc2ccccc2)C4(OCc3ccccc3)
AL-281/15328062	489.48	C26H23N3O7	CCOC(=O)c1ccc(cc1)NC(=O)C(=Cc2cccc(c2)[N+](=O)[O-])NC(=O)c3ccc(cc3)OC
AL-281/15328106	476.28	C20H18BrN3O6	OC(=O)CCCNC(=O)C(=Cc1cccc(c1)[N+](=O)[O-])NC(=O)c2cccc(c2)Br
AG-690/13771546	465.55	C23H19N3O4S2	NS(=O)(=O)c1ccc(cc1)NC(=O)CSc3nc(c2ccccc2)c(o3)c4ccccc4
AN-988/15291070	487.58	C26H25N5O3S	COc1ccc(cc1)NC(=O)CSc4nnc(c2cccc(c2)NC(=O)c3ccccc3(C))n4(C)
AL-281/15329237	459.34	C22H23BrN2O4	OC(=O)CCCCCNC(=O)C(=Cc1ccccc1)NC(=O)c2cccc(c2)Br
AN-988/40679900	487.56	C27H29N5O4	COCc3cc(C)nc(NN=Cc2ccc(OCC(=O)Nc1ccc(C)c(C)c1)c(c2)OC)c3(C#N)
AN-698/40704715	491.32	C20H15BrN2O6S	CN3C(=O)C(=Cc1cc(ccc1(OCC(O)=O))Br)SC3(=Nc2cccc(c2)C(O)=O)
AK-968/40733793	457.49	C19H15N5O5S2	CS(=O)(=O)c3ccc4nc(NC(=O)c1ccc(cc1)Cn2cc(cn2)[N+](=O)[O-])sc4(c3)
AN-919/41439801	442.52	C19H18N6O3S2	CCOC(=O)Cc4csc(NC(=O)CSc2nnc3c1ccccc1n(C)c3(n2))n4
AN-648/41665166	492.51	C25H20N2O7S	CCOc1ccc(cc1)NC(=O)CN4C(=O)C(=Cc2ccc(o2)c3cccc(c3)C(O)=O)SC4(=O)
AJ-292/41694909	494.6	C23H22N6O3S2	CCn1c(nnc1SCC(=O)Nc2ccc(cc2)S(=O)(=O)Nc3ccccn3)c4ccccc4
AK-968/41925551	452.49	C22H20N4O5S	Cc1cc(nc(C)n1)NS(=O)(=O)c2ccc(cc2)NC(=O)C=Cc3ccc4OCOc4(c3)
AQ-750/42210294	465.36	C21H18Cl2N2O4S	COc1cccc(c1)S(=O)(=O)NCc2ccc(cc2)C(=O)Nc3ccc(c(c3)Cl)Cl
AP-866/42442439	415.85	C20H14ClNO5S	O=C(O)c1cccc(c1)NC(=O)c2cc(ccc2Cl)S(=O)(=O)c3ccccc3
AG-690/11481937	489.35	C20H17BrN4O4S	COc3nccnc3(NS(=O)(=O)c1ccc(cc1)NC=CC(=O)c2ccc(cc2)Br)
AG-690/11959038	430.47	C22H22N8O2	O=C(CCCCC(=O)NN=Cc1[nH]c2ccccc2(n1))NN=Cc3[nH]c4ccccc4(n3)
AK-968/12163528	473.34	C21H14Cl2N4O3S	O=C(Nc1ccc(cc1)S(=O)(=O)Nc2cnc3ccccc3(n2))c4ccc(cc4Cl)Cl
AG-205/13459228	451.47	C25H25NO7	Cc1ccc(cc1)C(=O)C4=C(O)C(=O)N(CCCCCC(O)=O)C4(c2ccc3OCOc3(c2))
AM-807/13616313	487.63	C21H21N5O3S3	CCCSc3nnc(NC(=O)CSc1nc(ccc1(C#N))c2ccc(OC)c(c2)OC)s3
AE-848/34329056	495.54	C22H17N5O5S2	NS(=O)(=O)c1ccc(cc1)Nc4nc(c2ccc(cc2)[N+](=O)[O-])c(C(=O)Nc3ccccc3)s4
AF-399/15284064	494.55	C29H26N4O4	COc1ccc(cc1)C=5CC(c3cn(nc3(c2ccccc2))c4ccccc4)N(N=5)C(=O)CCC(O)=O
AG-205/07689009	478.53	C24H22N4O5S	Oc3ccccc3(C=NNC(=O)C(CSCc1ccccc1)NC(=O)c2ccc(cc2)[N+](=O)[O-])
AK-968/15607150	494.96	C24H19ClN4O4S	O=C(Nc1ccc(cc1)S(=O)(=O)Nc2ncccn2)c4cccc(COc3ccccc3Cl)c4
AG-690/09766009	488.41	C26H16O10	OC(=O)c1ccc(cc1(C(O)=O))Oc2ccc3ccc(cc3(c2))Oc4ccc(C(O)=O)c(c4)C(O)=O
AK-968/15255368	465.51	C25H27N3O6	COc3ccc(CC(=O)Nc2cccc(NC(=O)Cc1ccc(OC)c(c1)OC)n2)cc3(OC)
AF-399/15601312	493.63	C23H19N5O2S3	O=C(CSc4nnc(CSc2nc1ccccc1s2)n4(c3ccccc3))NCc5ccco5
AG-690/13153838	490.52	C26H26N4O6	COc1ccc(cc1)C(=O)NNC(=O)CCNC(=O)c3ccccc3(NC(=O)c2ccc(cc2)OC)
AQ-088/41085918	485.61	C27H27N5O2S	Cc1ccc(cc1)CNC(=O)CSc4nnc(c2cccc(c2)NC(=O)c3ccccc3(C))n4(C)
AK-968/12119137	448.48	C23H17FN4O3S	O=C(C=Cc1ccc(F)cc1)Nc2ccc(cc2)S(=O)(=O)Nc3cnc4ccccc4(n3)
AG-205/08399012	455.56	C24H33N5O4	COc1ccc(cc1)NCC(=O)NN=Cc2cc(ccc2N(CC(C)C)CC(C)C)[N+](=O)[O-]
AG-690/09674015	492.66	C30H40N2O4	O=C(c1ccccc1)C(CCCCC(CN2CCOCC2)C(=O)c3ccccc3)CN4CCOCC4
AG-690/10023013	444.51	C24H20N4O3S	Cc1ccnc(n1)NS(=O)(=O)c2ccc(cc2)NC(=O)c3ccc(cc3)c4ccccc4
AG-690/12244642	472.52	C25H20N4O4S	Cc1ccnc(n1)NS(=O)(=O)c2ccc(cc2)NC(=O)c3ccc(cc3)C(=O)c4ccccc4
AG-690/12557117	476.49	C20H16N2O8S2	OC(=O)CCCC(C(O)=O)N3C(=O)C(=Cc1ccc(o1)c2ccc(cc2)[N+](=O)[O-])SC3=S
AF-962/31930018	470.38	C18H20N2O7P2S	OC(CCN2C(=CSC2(=Nc1ccccc1))c3ccccc3)(P(O)(O)=O)P(O)(O)=O

AN-329/42158848	483.61	C24H25N3O4S2	COc1ccc(cc1)CC(=O)NC(Nc2ccc(cc2)S(=O)(=O)NCCc3ccccc3)=S
AK-968/14196291	490.65	C20H18N4O3S4	CCOc3ccc4nc(SCC(=O)NNC(=O)CSc2nc1ccccc1s2)sc4(c3)
AN-465/43369584	483.4	C25H24Cl2N4O2	COc2cc(ccc2(OCc1ccc(nc1)Cl))CNCCNc3ccnc4cc(ccc34)Cl
AP-970/43253453	404.54	C17H16N4O2S3	CCCC(=O)Nc2nc1ccc(cc1s2)NC(NC(=O)c3cccs3)=S
AN-465/43411315	381.43	C22H23NO5	COc3ccc(CCNCc1ccc(o1)c2ccc(cc2)C(=O)O)cc3(OC)
